# Commercially available activity monitors such as the fitbit charge and apple watch show poor validity in patients with gait aids after total knee arthroplasty

**DOI:** 10.1186/s13018-024-04892-9

**Published:** 2024-07-15

**Authors:** Paul Kooner, Sandhya Baskaran, Vanessa Gibbs, Sam Wein, Ronald Dimentberg, Anthony Albers

**Affiliations:** 1 Division of Orthopaedic Surgery, McGill University, McGill University Health Centre, 1650 Cedar Avenue, Montreal, Qc H3G 1A4 Canada; 2 Montreal General Hospital, Division of Orthopaedic Surgery, McGill University, Montreal, Canada; 3 Division of Orthopaedic Surgery, St. Mary’s Hospital, McGill University, Montreal, Canada

**Keywords:** Activity monitor, Step count, Fitbit, Apple watch, Total knee arthroplasty

## Abstract

**Purpose:**

The aim of this study is to determine the validity of consumer grade step counter devices during the early recovery period after knee replacement surgery.

**Methods:**

Twenty-three participants wore a Fitbit Charge or Apple Watch Series 4 smart watch and performed a walking test along a 50-metre hallway. There were 9 males and 14 females included in the study with an average age of 68.5 years and BMI of 32. Each patient wore both the Fitbit Charge and Apple Watch while completing the walking test and an observer counted the ground truth value using a thumb-push tally counter. This test was repeated pre-operatively with no gait aid, immediately post operatively with a walker, at 6 weeks follow up with a cane and at 6 months with no gait aid. Bland-Altman plots were performed for all walking tests to compare the agreement between measurement techniques.

**Results:**

Mean overall agreement of step count for pre-operative and at 6 months for subjects walking without gait aids was excellent for both the Apple Watch vs. actual and Fitbit vs. actual with bias values ranging from − 0.87 to 1.36 with limits of agreement (LOA) ranging between − 10.82 and 15.91. While using a walker both devices showed extremely little agreement with the actual step count with bias values between 22.5 and 24.37 with LOA between 11.7 and 33.3. At 6 weeks post-op while using a cane, both the Apple Watch and Fitbit devices had a range of bias values between − 2.8 and 5.73 with LOA between − 13.51 and 24.97.

**Conclusions:**

These devices show poor validity in the early post operative setting, especially with the use of gait aids, and therefore results should be interpreted with caution.

## Introduction

The need for joint replacement surgery continues to rise with over 100 000 total knee arthroplasty (TKA) surgeries performed in Canada in 2021-22 and 150 000 patients still awaiting surgery [1]. With increasing demands and wait times for surgery, patients are experiencing a greater decline in function and physical activity levels. While traditional clinical outcome measures of function and patient satisfaction after total joint replacement has relied on subjective patient reported outcome measures (PROMs), the growing popularity of commercially available wearable activity monitors such as the Apple Watch (Apple, Cupertino, CA, USA) or Fitbit Charge (Fitbit, San Francisco, CA, USA) has provided a novel way to objectively monitor patient’s recovery after surgery. With post-acute care costs of lower limb arthroplasty accounting for 36% of total costs, a remotely based objective monitoring method such as tracking step count is an attractive option to monitor the patient’s post-operative course [2]. With the growing demand of joint replacement surgery, limiting these costs while improving patient outcomes continues to be a central focus of arthroplasty research.

Consumer grade wearable step counter devices such as the Apple Watch or Fitbit Charge are inexpensive tools that enable patients, researchers, and clinicians to objectively monitor physical activity. These devices have demonstrated a high inter- and intra-device reliability that has provided clinicians with a valid and reliable assessment tool in healthy populations with normal gait patterns [3–5]. However, there is a paucity of literature investigating those with chronic physical impairments or acutely after surgery [6]. Patients with traumatic brain injury or stroke have poor reliability with these devices as they have altered gait mechanics and slower walking speeds [7–10]. In contrast to research specific devices, the StepWatch Activity Monitor (Orthocare Innovations, Oklahoma City, OK, USA) has shown higher accuracy in this population, however this device is primarily used in the research setting and is impractical compared to commercially available devices [9, 10]. It is unclear how the use of gait aids or rehabilitation after joint replacement surgery impacts the accuracy of these devices.

The aim of this study is to determine the validity of consumer grade commercially available step counter devices during the early recovery period after knee replacement surgery. To date there has been no validation study investigating the reliability of these devices in the pre- and post-operative setting. Nonetheless, there are some applications on the market which aim to help physicians monitor their patient’s progress using this technology [11, 12]. With the growing trend of smartphone-based apps available to monitor recovery, determining their dependability may allow us to better understand the role of wearable activity monitors as clinically applicable tools for benchmarking results after joint arthroplasty. In particular, the accuracy of these devices at slower walking speeds or with gait aids such as canes or walkers. We hypothesize that due to the change in walking mechanics with gait aids and decreased walking speeds, that consumer grade activity trackers will underestimate step count.

## Methods

Twenty-three participants wore a Fitbit Charge (Fitbit, San Francisco, CA, USA) or Apple Watch Series 4 (Apple, Cupertino, CA, USA) smart watch and performed a walking test along a 50-metre hallway. Each patient wore both the Fitbit Charge and Apple Watch while completing the walking test. These devices were provided by the research team. An observer counted the ground truth value using a thumb-push tally counter while the readings from the devices communicated with the smartphone application on a handheld device. The same team of research assistants (Authors: SB, VG, SM) performed all walking tests to record the ground truth values to ensure consistency in data collection. This data was recorded at the end of each walking test and each participant performed the walking test twice while taking the mean step count and time of the two tests. This test was repeated pre-operatively 4–6 weeks prior to surgery with no gait aid, immediately post operatively (days 1–3) with a walker, at 6 weeks follow up with a cane and at 6 months with no gait aid. Inclusion criteria are: Adults (> 18 years old) who required a primary total knee arthroplasty. Patients were excluded if they had previous joint replacement surgery, revision surgery, body mass index (BMI) > 45 kg/m^2^, or a diagnosis other than osteoarthritis. There were 9 males and 14 females included in the study with an average age of 68.5 years and BMI of 32. Bland-Altman plots were performed for each test to compare the agreement between measurement techniques for each individual (Fitbit vs. actual step count and Apple Watch vs. actual step count). The dashed lines represent 95% limits of agreement (LOAs). Comparisons were made using ANOVA for each test with 80% power to detect differences of more than one step between measurement methods, assuming α = 0.05. This study was approved by the institutional ethics review board.

## Results

**Fig. 1:**
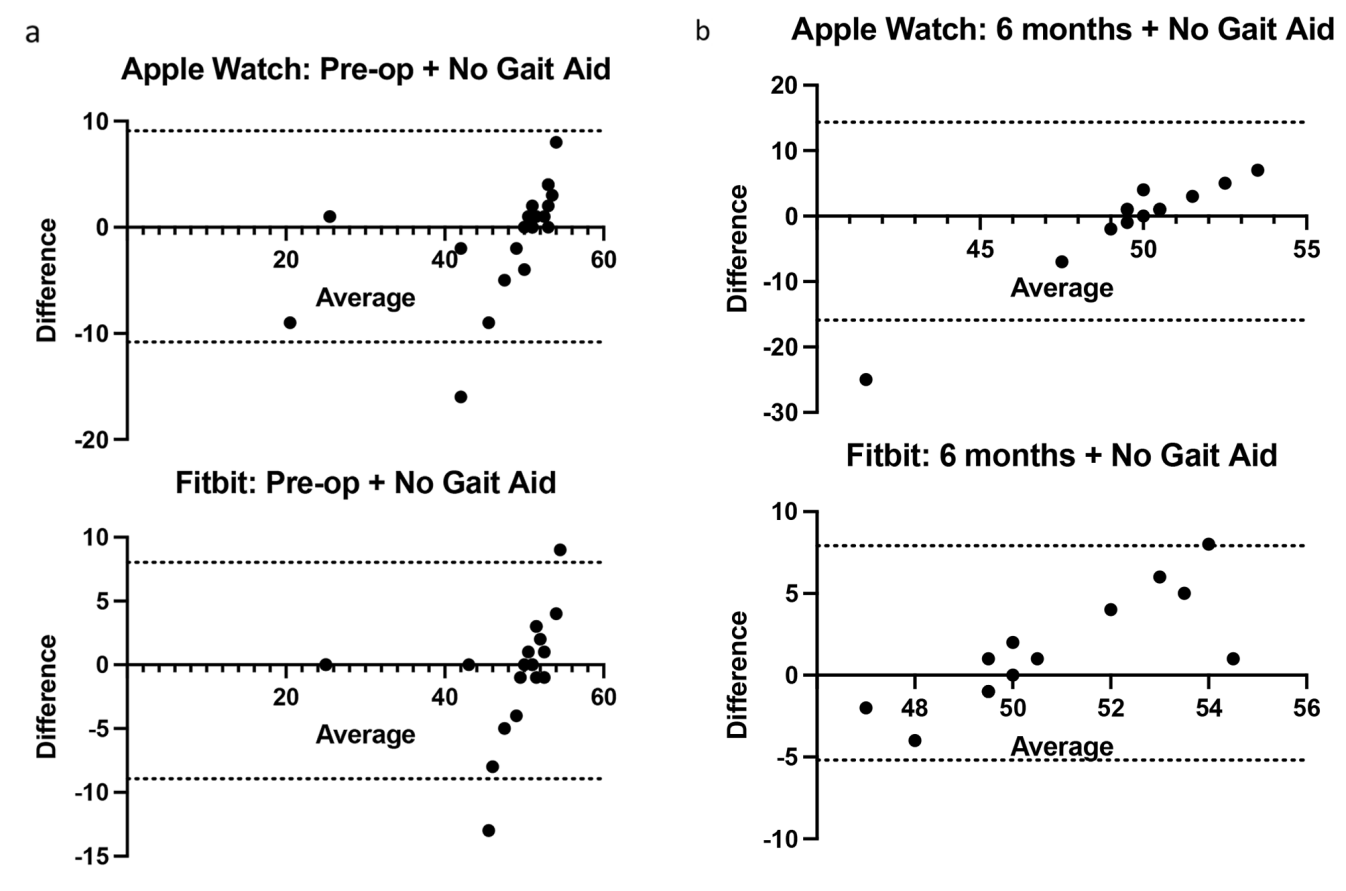
Bland Altman plots for patients with no gait aid for 23 participants with Apple Watch and Fitbit Charge with (**a**) no gait aid at pre operative assessment (**b**) no gait aid at final 6 months post operative assessment

**Fig. 2:**
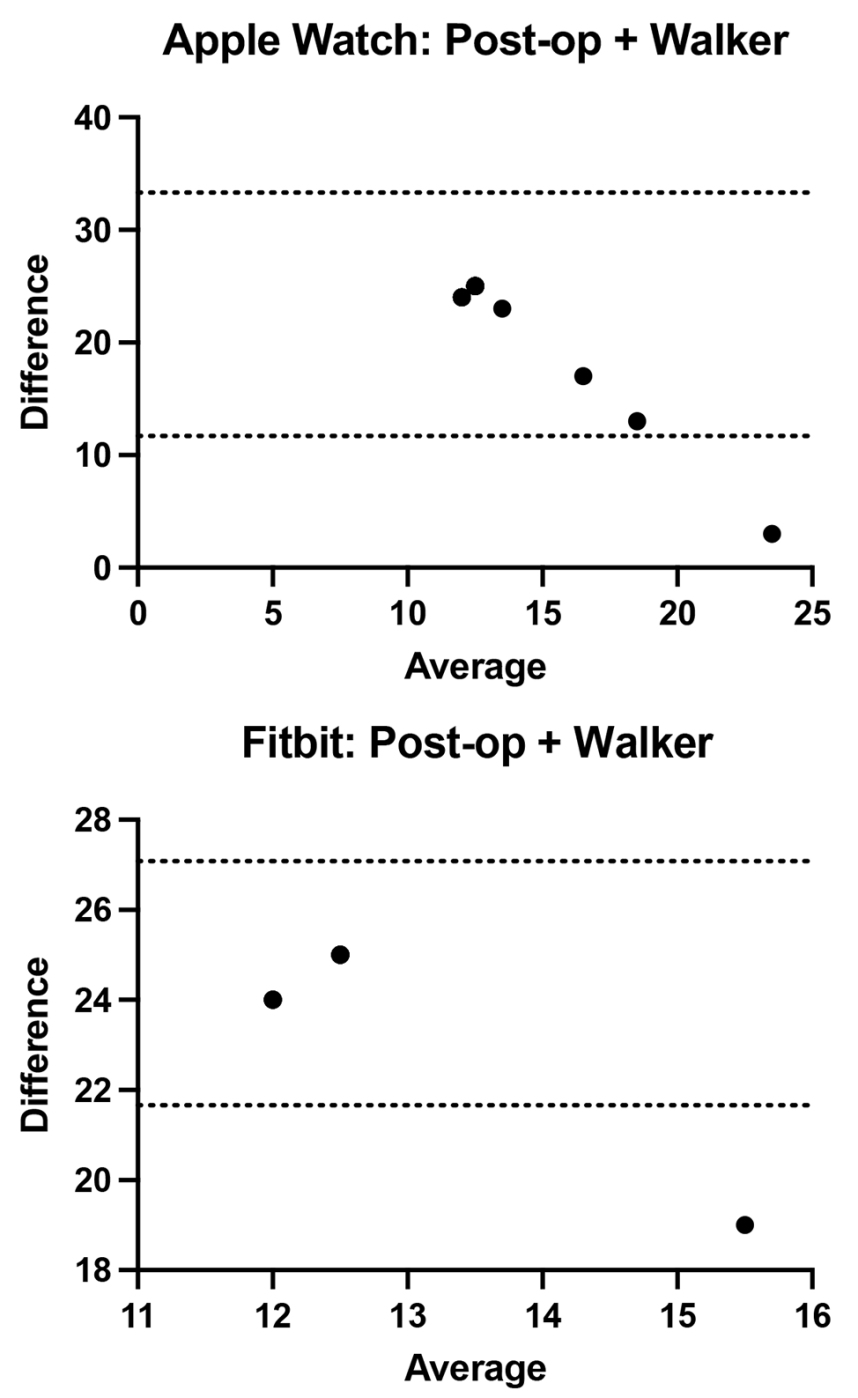
Bland Altman plots immediate post operatively (Days 1–3) for 23 participants with (**a**) Apple Watch with walker at immediate post operative assessment (**b**) Fitbit Charge with walker at immediate post operative assessment

**Fig. 3:**
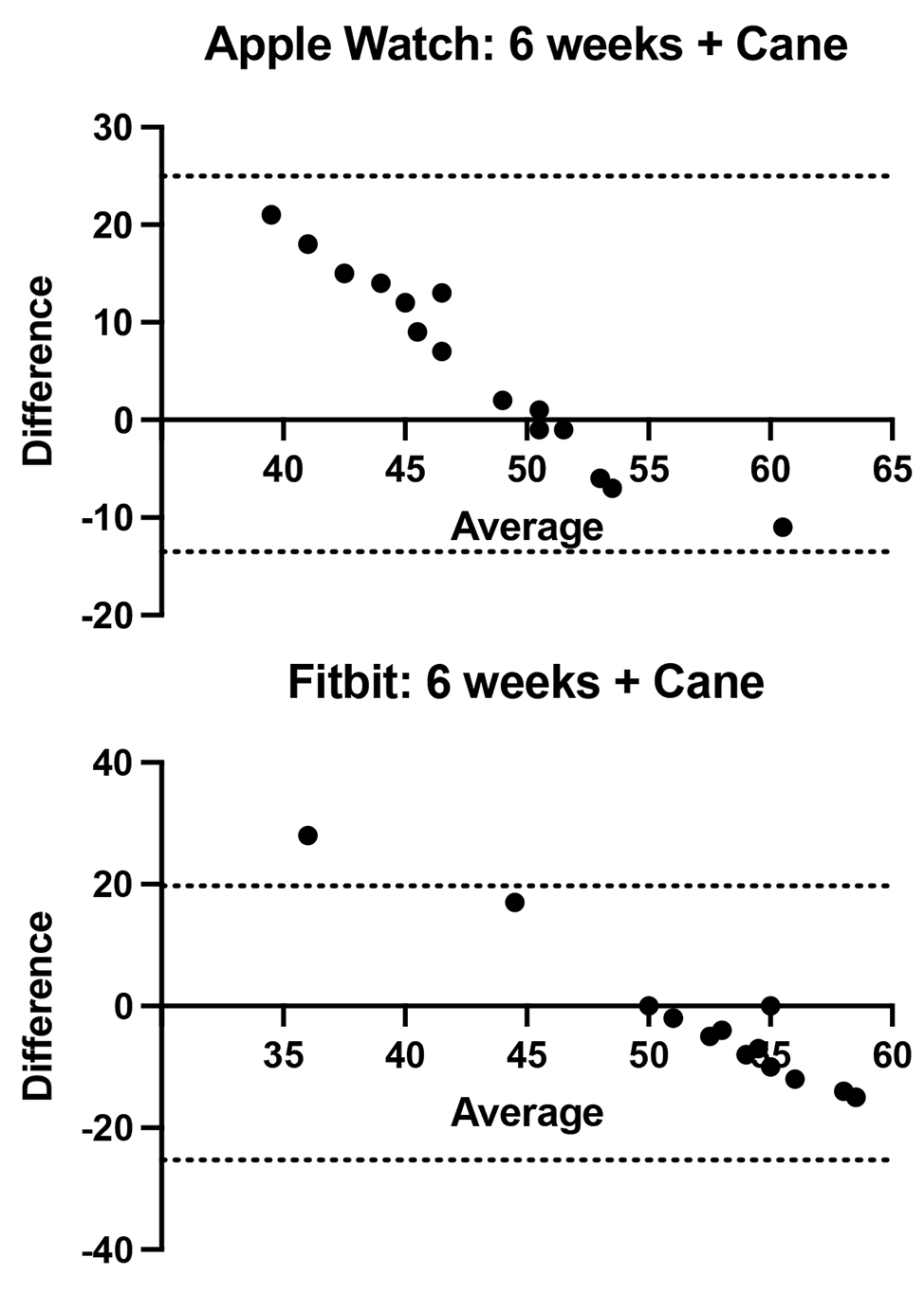
Bland Altman plots at 6 weeks post operatively for 23 participants with (**a**) Apple Watch with cane at 6 weeks post operative assessment (**b**) Fitbit Charge with cane at 6 weeks post operative assessment

The overall agreement for each device and walking test are shown in Table 1. Mean overall agreement for pre-operative and at 6 months for subjects walking without gait aids was excellent for both the Apple Watch vs. actual and Fitbit vs. actual with bias values ranging from − 0.87 to 1.36 with limits of agreement (LOA) ranging between − 10.82 and 15.91 (Fig. 1a and b). While using a walker in the immediate post op setting, both devices showed extremely little agreement with the actual step count with bias values between 22.5 and 24.37 with LOA between 11.7 and 33.3 illustrating exceptionally unreliable step counts (Fig. 2). Furthermore, limited data was captured due to many devices recording zero step count for the participant. At 6 weeks post-op while using a cane, the Bland-Altman plots for both the Apple Watch and Fitbit devices had a range of bias values between − 2.8 and 5.73 with LOA between − 13.51 and 24.97 (Fig. 3).

**Table 1:** Agreement between activity monitors and true step count

Test (step count)	Bias	SD of Bias	LOA Lower (95% CI)	LOA Upper (95% CI)
Apple Pre-op vs. Actual	-0.87	5.01	-10.82	9.08
Fitbit Pre-op vs. Actual	-0.46	4.33	-8.94	8.03
Apple Post-op vs. Actual	22.5	5.51	11.7	33.3
Fitbit Post-op vs. Actual	24.37	1.38	21.66	27.08
Apple 6 weeks vs. Actual	5.73	9.82	-13.51	24.97
Fitbit 6 weeks vs. Actual	-2.8	11.48	-25.31	19.71
Apple 6 months vs. Actual	-0.79	7.72	-15.91	14.34
Fitbit 6 months vs. Actual	1.36	3.34	-5.19	7.91

**Fig. 4:**
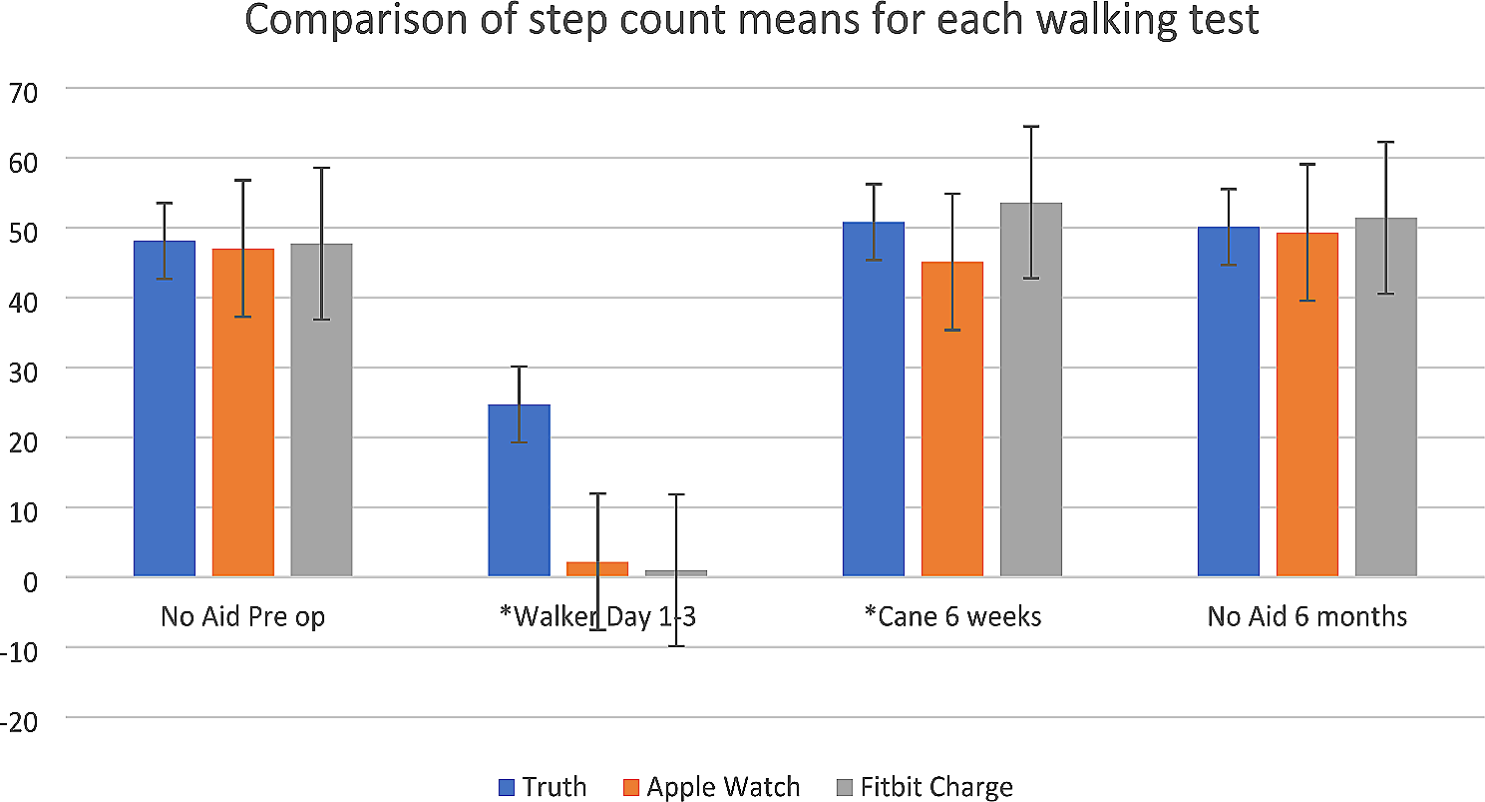
Comparison of step count means. *Statistically significant difference of means from ground truth value

The four walking tests and the comparison of mean step counts for each device with the actual step count are shown in Fig. 4. In the early post operative period the mean step count of both the Apple Watch and Fitbit (2.2 +/- 5.6; 0.9 +/- 3.0 respectively) were significantly different from the mean ground truth value (24.7 +/- 0.4) (*p* < 0.0001). Again, at 6 weeks post op both the Apple Watch and Fitbit (45.1 +/- 10.3; 53.6 +/- 11.3 respectively) had significantly different values from the actual step count (50.8 +/- 1.4) (*p* = 0.036). At both pre-operative visit and 6 months follow up the mean step count was comparable to the actual count values as seen in Fig. 4.

## Discussion

Currently there is a paucity of literature to validate consumer grade wearable activity monitors as proper measurements of physical activity levels in patients with TKA during the post operative period. In this study, both step count devices demonstrated an unacceptable error rate immediately after surgery and up to 6 months post operatively. Once patient functional status improved, and they no longer required the use of gait aids, the devices showed better agreement to the actual step count values. These results show that we must interpret this data with caution if we are to rely on remotely based objective monitoring systems that allow us to connect with patients and track recovery in the post operative setting.

The inconsistency in agreement is likely due to the altered gait mechanics and slower walking speeds with gait aids that is predominant in the early post operative setting. These activity monitors rely on an acceleration threshold algorithm and accelerometers to quantify step count [11]. Throughout studies in cardiology, neurology, and geriatrics, accelerometer-based devices have shown inconsistency in accuracy when compared to the general population [7, 8, 13–16]. The irregularity in gait patterns while using a walking aid may influence step counts of wrist worn devices as the traditional swing of the arm during the gait cycle is impaired and the accelerometer is unable to detect steps taken. This is particularly true with the use of a walker as little to no step counts were captured as shown by the minimal points on our Bland Altmann plots (Fig. 2). When these devices are compared to research specific devices such as the Stepwatch Activity Monitor, the gait speed may be calibrated for each individual, regardless of baseline ambulatory status or gait pattern [10]. It incorporates a custom sensor that allows for this calibration and shows an accuracy of 98.6% in patients after stroke with altered gait mechanics and slower walking speeds. When compared to our results, only at pre-operative and post-operative assessment with no gait aid did these consumer grade devices show a difference of less than 3 steps to the ground truth value (Fig. 4). Furthermore, the location of the device has been shown to influence outcomes, as studies show that the contralateral hip or ankle is the more reliable measure, even at slower walking speeds [17, 18]. A systematic review and a direct comparison of ankle versus waist worn devices found that at slower walking speeds these devices should be worn at the ankle to estimate step count [19, 20]. However, this may be impractical for patients as these devices were intended to be wrist-worn by the manufacturer and may influence compliance and results.

There was an increasing proportional bias seen in average step count as the patients began to move with increasing acceleration from a stationary position. The Bland-Altman plots suggested a trend towards increased agreement between step count and ground truth values especially in Fig. 3 while patients ambulated with a cane. This may reflect a change in agreement as the patient begins to increase acceleration and may alter the results if a longer or continuous period of monitoring was used compared to our 50-meter track. However, in the early post operative period and at 6 weeks, fatigue in patient’s walking ability became a concern as patients were often tired after each walking test. A longer or continuous monitoring period may allow us to further assess the endurance or stamina of physical activity levels in patients and may be a future direction of research.

The clinical impact of these devices has grown tremendously in the past several years, as the development of smartphone-based apps or platforms have allowed clinicians to monitor recovery remotely. In two RCTs, self-directed rehabilitation with a wearable device linked to a smartphone app showed non-inferiority to standard in person rehabilitation protocols after TKA [11, 12]. Furthermore, step count goal setting of these devices and increased feedback can significantly improve outcomes and has led to decreased rehospitalization and opioid use in the 2 weeks following TKA [21–24]. This illustrates the profound potential of these devices to optimize recovery, provide feedback to clinicians and to possibly isolate those individuals who are lacking behind clinical benchmarks. However, our validation study shows that there is still room for improvement, and that we must acknowledge the limitations of these devices before implementing clinical decision making or as a research tool.

Future considerations for the use of these devices include components such as patient engagement platforms, accurate devices at slower speeds and providing live feedback and step count monitoring to set patient-specific benchmarks for recovery. Currently, spatial-temporal gait parameter sensors or gait kinematic tracking is under development which could allow better monitoring of patient recovery kinematics following surgery [25, 26]. The use of smart implants which are implantable devices are currently under development that will allow real time feedback of patients gait behavior, range of motion and step count [27, 28]. Implementation of this technology has the potential to offer new insights for clinical research as it allows clinicians to detect variations in gait patterns, speed in recovery and return to function.

There were limitations to this study. Due to the limited sample size and the use of only two devices, these results may not be generalizable to all patients or all commercially available monitors. Nevertheless, we felt that our analysis and results were sound and reflected the findings of current literature. We chose the two most common devices available on the market for analysis at the time of study. Furthermore, there were no of levels of agreement that were deemed acceptable or unacceptable in the accuracy of measurements of step count. This may influence interpretation of the data and future research should include what are acceptable levels of agreement for these smart devices. Collecting data from a continuous monitoring perspective and broader patient population post operatively could help illustrate when step counters become more accurate.

The market for commercially available monitoring devices and smartphone apps is rapidly evolving in total joint arthroplasty, however clinicians should be aware of their limitations at this time. Our results show that these devices have a poor validation of step count in the early post operative setting, especially with the use of gait aids, and therefore results should be interpreted with caution. Future development of these devices could provide exciting new technology that will allow clinicians and researchers to better monitor recovery and improve patient outcomes.

## Data Availability

No datasets were generated or analysed during the current study.

## References

[1] Canadian Institute for Health Information (2020). Hip and knee replacements in Canada: CJRR Annual statistics Summary, 2021–2022.

[2] Bozic KJ, Ward L, Vail TP, Maze M (2014). Bundled payments in total joint arthroplasty: targeting opportunities for quality improvement and cost reduction. Clin Orthop Relat Res.

[3] Kooiman TJ, Dontje ML, Sprenger SR, Krijnen WP, van der Schans CP, de Groot M (2015). Reliability and validity of ten consumer activity trackers. BMC Sports Sci Med Rehabil.

[4] Alharbi M, Bauman A, Neubeck L, Gallagher R (2016). Validation of fitbit-flex as a measure of free-living physical activity in a community-based phase III cardiac rehabilitation population. Eur J Prev Cardiol.

[5] Brickwood KJ, Watson G, O’Brien J, Williams AD. (2019) Consumer-based wearable activity trackers increase physical activity participation: systematic review and meta-analysis. JMIR mHealth uHealth, 7 e11819. 10.2196/11819 PMC6484266 30977740

[6] Schmalzried TP, Shepherd EF, Dorey FJ, Jackson WO, dela Rosa M, Fa’ave F, McKellop HA, McClung CD, Martell J, Moreland JR, Amstutz HC (2000). The John Charley Award. Wear is a function of use, not time. Clin Orthop Relat Res.

[7] Fulk GD, Combs SA, Danks KA, Nirider CD, Raja B, Reisman DS (2013). Accuracy of 2 activity monitors in detecting steps in people with stroke and traumatic brain injury. Phys Ther.

[8] Schaffer SD, Holzapfel SD, Fulk G, Bosch PR (2017). Step counter accuracy and reliability of two activity tracking devices in people after stroke. Physiother Theory Pract.

[9] Macko RF, Haeuber E, Shaughnessy M (2002). Microprocessor-based ambulatory activity monitoring in stroke patients. Med Sci Sports Exerc.

[10] Mudge S, Stott NS, Walt SE (2007). Criterion validity of the stepwatch activity monitor as a measure of walking activity in patients after stroke. Arch Phys Med Rehabil.

[11] Crawford DA, Duwelius PJ, Sneller MA, Morris MJ, Hurst JM, Berend KR, Lombardi AV (2021). Mark Coventry Award: Use of a smartphone-based care platform after primary partial and total knee arthroplasty: a prospective randomized controlled trial. Bone Joint J.

[12] Tripuraneni KR, Foran JRH, Munson NR, Racca NE, Carothers JT (2021). A smartwatch paired with a mobile application provides postoperative self-directed rehabilitation without compromising total knee arthroplasty outcomes: a randomized controlled trial. J Arthroplasty.

[13] Thorup C, Andreasen J, Sørensen E, Grønkjær M, Dinesen B, Hansen J (2017). Accuracy of a step counter during treadmill and daily life walking by healthy adults and patients with cardiac disease. BMJ Open.

[14] Pitta F, Troosters T, Spruit MA, Decramer M, Gosselink R (2005). Activity monitoring for assessment of physical activities in daily life in patients with chronic obstructive pulmonary disease. Arch Phys Med Rehabil.

[15] Langer D, Gosselink R, Sena R, Burtin C, Decramer M, Troosters T (2009). Validation of two activity monitors in patients with COPD. Thorax.

[16] Larkin L, Nordgren B, Purtill H, Brand C, Fraser A, Kennedy N. Criterion validity of the activPAL activity monitor for sedentary and physical activity patterns in people who have rheumatoid arthritis. Phys Ther; 2015. 10.2522/ptj.20150281 26637646

[17] Goel R, Danoff JR, Blevins K, Purtill JJ, Chen AF (2020). A step in the right direction: body location determines activity tracking device accuracy in total knee and hip arthroplasty patients. J Am Acad Orthop Surg.

[18] Kooner P, Schubert T, Howard JL, Lanting BA, Teeter MG, Vasarhelyi EM (2021). Evaluation of the effect of gait aids, such as canes, crutches, and walkers, on the accuracy of step counters in healthy individuals. Orthop Res Reviews.

[19] Feehan LM, Geldman J, Sayre EC, Park C, Ezzat AM, Yoo JY, Hamilton CB, Li LC (2018). Accuracy of Fitbit devices: systematic review and narrative of synthesis of quantitative data. JMIR Mhealth Uhealth.

[20] Karabulut M, Crouter SE, Bassett DR Jr. Comparison of two waist-mounted and two ankle-mounted electronic pedometers (2005). Eur J Appl Physiol, 95(4):335–43. 10.1007/s00421-005-0018-3 16132120

[21] van Dijk-Huisman HC, Weemaes ATR, Boymans TAEJ, Lenssen AF, de Bie RA (2020). Smartphone app with an accelerometer enhances patients’ physical activity following elective orthopedic surgery: a pilot study. Sensors.

[22] Mehta SJ, Hume E, Troxel AB, Reitz C, Norton L, Lacko H, McDonald C, Freeman J, Marcus N, Volpp KG (2020). Effect of remote monitoring on discharge to home, return to activity, and rehospitalization after hip and knee arthroplasty: a randomized clinical trial. JAMA Netw Open.

[23] Pronk Y, Peters MCWM, Sheombar A, Brinkman JM (2020). Effectiveness of a mobile ehealth app in guiding patients in pain control and opiate use after total knee replacement: randomized controlled trial. JMIR mHealth uHealth.

[24] Van der Walt N, Salmon LJ, Gooden B, Lyons MC, O’Sullivan M, Martina K, Pinczewski LA, Roe JP (2018). Feedback from activity trackers improves daily step count after knee and hip arthroplasty: a randomized controlled trial. J Arthroplasty.

[25] Renani MS, Myers CA, Zandie R, Mahoor MH, Davidson BS, Clary CW (2020). Deep learning in gait parameter prediction for OA and TKA patients wearing IMU sensors. Sensors.

[26] Youn IH, Youn JH, Zeni JA, Knarr BA (2018). Biomechanical gait variable estimation using wearable sensors after unilateral total knee arthroplasty. Sensors.

[27] https://www.prnewswire.com/news-releases/zimmer-biomet-and-canary-medical-announce-fda-de-novo-classification-grant-and-authorization-to-market-the-worlds-first-and-only-smart-knee-implant-301364874.html.

[28] Cyarto EV, Myers AM, Tudor-Locke C (2004). Pedometer accuracy in nursing home and community-dwelling older adults. Med Sci Sports Exerc.

